# FZD7 accelerates hepatic metastases in pancreatic cancer by strengthening EMT and stemness associated with TGF-β/SMAD3 signaling

**DOI:** 10.1186/s10020-022-00509-1

**Published:** 2022-07-19

**Authors:** Zhongbo Zhang, Yuanhong Xu

**Affiliations:** Department of Pancreatic and Biliary Surgery, The First Hospital of China Medical University, 155 Nanjing North Street, Heping, Shenyang, 110001 Liaoning People’s Republic of China

**Keywords:** FZD7, EMT, Wnt pathway, Pancreatic cancer, Hepatic metastases

## Abstract

**Background:**

Metastasis of malignant tumors accelerates systemic failure and hastens the deaths of pancreatic cancer patients. During the metastatic process, the physical translocation of cancer cells from the primary lesion to distant organs and is crucial. CSCs properties, such as self-renewal and multiple-direction differentiation capacity are essential for colonization in the microenvironment of distant organs and metastatic lesion formation. It is widely believed that EMT can cause cancer cells to penetrate blood vessels by undergoing phenotypic and cytoskeletal changes, so that they can infiltrate surrounding tissue and disseminate from the primary tumor to the blood circulation, where they are termed circulating tumor cells (CTCs), while CTCs often exhibit stemness properties. Accumulating evidence demonstrates that some EMT-related transcription factors are essential for CSCs self-renewal, so cancer cells that have undergone EMT typically acquire increased stemness properties. Abnormal activation of the WNT signaling pathway can drive a series of gene transcripts to promote EMT in multiple types of cancer, and among different Frizzled receptors of WNT signaling pathway, FZD7 expression is associated with distant organ metastasis, advanced clinical stages, and poor clinical prognosis. Objective of this study is to demonstrate that high FZD7 expression in pancreatic cancer can accelerate hepatic metastases and elucidate the related molecular mechanisms.

**Methods:**

The expression of Frrizled receptor 7 (FZD7) in pancreatic ductal adenocarcinoma (PDAC) and relating survival rate were analyzed by bioinformatics, histochemistry assay and follow-up study. In vitro, FZD7 expression was silenced by lentiviral vectors carrying short hair RNA (shRNA) or upregulated by overexpression plasmid. Then, Wound-healing and Transwell experiment was used to analyze the abilities of migration and invasion; the levels of epithelial-to-mesenchymal transition (EMT) relating phenotype proteins, stemness relating phenotype proteins, and signaling molecular proteins were measured by Western-blot; cell stemness was evaluated by sphere forming ability of cells in suspension culture and detecting the proportion of CD24^+^CD44^+^ cells with flow cytometry. TGF-β1 was used to induce EMT, and observe the effect of shRNA silencing FZD7 on which.

**Results:**

High level of FZD7 expression in pancreatic cancer samples was associated with earlier hepatic metastasis. In vitro upregulation FZD7 can enable pancreatic cancer cells to obtain stronger migration and invasion ability and higher mesenchymal phenotype, and vice versa; the proportion of cancer stem cell (CSC) was also positively correlated with the level of FZD7; cells forming spheres in suspension culture showed stronger migration and invasion ability and higher level of mesenchymal phenotype than normal adherent cultured cells; the level of FZD7 was positively correlated with the level of activated β-catenin. Silencing FZD7 expression can attenuate EMT induced by TGF-β1 stimulating, and TGF-β1 stimulating can also upregulate stemness phenotype expression, such as ABCG2, CD24, and CD44 by mediating of FZD7.

**Conclusions:**

High FZD7 expression in pancreatic cancer can accelerates hepatic metastases by promoting EMT and strengthening cell stemness, and FZD7 can work through the canonical Wingless-type (WNT) signaling pathway and participate in TGF-β/SMAD3 signaling pathway also.

## Background

The overall survival rate for pancreatic cancer is only 8% (Siegel et al. 2016). Metastasis of malignant tumors accelerates the process of systemic failure and hastens the deaths of patients. Early pancreatic cancer is usually asymptomatic; thus, the majority of pancreatic cancer cannot undergo radical surgery, because liver metastasis or other advanced manifestations of malignancy had already occurred at the time when patients were initially diagnosed.

During the metastatic process, the physical translocation of cancer cells from the primary lesion to distant organs and colonization in the microenvironment is crucial (Chaffer and Weinberg 2011). It is widely believed that procedure of EMT enables cancer cells to acquire the ability to infiltrate surrounding tissue and metastasize to distant organs via the circulatory blood (Chaffer et al. 2016). EMT can cause tumor cells to penetrate blood vessels by undergoing phenotypic and cytoskeletal changes, so that they can disseminate from the primary tumor to the blood circulation, where they are termed circulating tumor cells (CTCs) (Pantel and Brakenhoff 2004; Guarino 2007; Ksiazkiewicz et al. 2012).

In addition, CSCs with self-renewal and multiple-direction differentiation capacity are essential for successful metastatic lesion formation also (Lau et al. 2017). However, it is interesting that CTC subsets often exhibit many stemness properties (Gkountela et al. 2019), which may facilitate the colonization of distant organs and development of metastatic lesions. Cancer cells that have undergone EMT typically acquire increased stemness properties, because some EMT-related transcription factors, such as Snail and Zeb1, are essential for CSCs self-renewal (Lau et al. 2017). EMT activator Zeb1 indirectly enhances cell stemness by inactivating microRNA that can inhibit cell stemness (Wellner et al. 2009).

Accumulating evidence demonstrates that abnormal activation of the WNT signaling pathway can drive a series of gene transcripts to promote EMT in multiple types of cancer (Anastas and Moon 2013). Increased levels of Frizzled receptors displayed in multiple types of cancers imply that abnormal activation of WNT signaling accelerates cancer progression (Gurney et al. 2012). Among different Frizzled receptors, it has been reported that FZD7 levels are associated with distant organ metastasis, advanced clinical stages, and poor clinical prognosis. For example, in gastric cancer cells (Li et al. 2018) and esophageal cancer (Cao et al. 2017), high levels of FZD7 serve as important mediators of EMT and can promote surrounding tissue invasion and distant organ metastasis. Downregulation of FZD7 expression significantly attenuated the invasion and metastatic abilities of colon cancer cells (Ueno et al. 2009). Recent studies have shown that in cervical and ovarian cancers, downregulation of FZD7 was accompanied by a decreased expression of vimentin and Snail and increased E-cadherin expression, and tumours presented an attenuated ability to invade and migrate (Deng et al. 2015; Asad et al. 2014).

To investigate the role of FZD7 in pancreatic cancer, we investigated high level of FZD7 expression in PDAC tissue sample firstly, then demonstrated that high levels of FZD7 expression associated with earlier hepatic metastasis in PDAC patients, and high FZD7 expression can promote EMT and CSC amplification in vitro; we also confirmed the correlation between CSC and EMT, and the synergistic effect of FZD7 with TGF-β/SMAD3, so explained the mechanism of FZD7 high expression promoting hepatic metastasis.

## Methods and materials

### Bioinformatics

The Oncomine Cancer Microarray database (http://www.oncomine.org) was used to study gene expression of FZD7 in pancreatic cancer tissue samples, and to perform comparison analysis on levels of various Frizzled receptors in pancreatic adenocarcinoma. Correlation analysis was performed on the R2 microarray analysis and visualization platform (http://r2.amc.nl).

### Pancreatic cancer tissue samples and Computerized Tomography (CT) images

The human cancer tissue samples and CT images included in this study were obtained from Department of Pancreatic-Biliary Surgery in the First Hospital of China Medical University, and their use for scientific research was approved by the hospital ethics committee. A total of 42 samples of pancreatic cancer tissues and paracancerous normal pancreatic tissues were collected from the end of 2015 until the beginning of 2021. According to the American Joint Committee on Cancer Staging (AJCC) pancreatic cancer staging standard 8th edition, all patients were classified as Tumor Node Metastasis (TNM) stage Ia–IIb and had undergone radical resection. Each patient was diagnosed with PDAC by two senior pathologists. Excluding patients lost to follow-up (whose outcomes remain unknown), all the other patients succumbed to advanced pancreatic cancer during the follow up period, without any other cause of death or accidental death. The clinicopathological information of patients and their relationship with the pathological results are shown in Table 1.

**Table 1:** The relationship between Fzd7 and basic clinical information of PDAC patients

	Samples	Fzd7 expression n (%)		
	(n)	Negative	Positive	*P* value^a,b^
Sex				0.292
Male	21	7	14	
Female	21	4	17	
Age^c^				0.867
≦ 55	20	5	15	
> 55	22	6	16	
TNM stage (AJCC)				0.116
I	20	3	17	
II	22	8	14	

^a^X^2^ test; ^b^Significant, *P* < 0.05; ^c^Median age

### Immunohistochemistry (IHC) staining

Informed consent was signed before surgery to obtain consent from family members and patients to use part of cancer tissue sample for scientific research experiments. After the cancer tissue sample was removed from the body, it was immediately stored in paraformaldehyde and immunohistochemical staining was performed within 3 days.

Immunohistochemistry staining was performed using the standard streptavidin–biotin–peroxidase complex method. Briefly, 4–6 μm paraffin sections were dewaxed and rehydrated using xylol and a descending series of alcohol solutions. Endogenous peroxidase activity was blocked using 3% hydrogen peroxide for 20 min. Sections were heated in a microwave oven for 10 min in 10 mM citrate buffer for antigen retrieval (pH 6.0). Sections were subsequently incubated with primary antibodies overnight at 4 °C (anti-FZD7, 1:500, Abcam, cat. no. ab64636, Cambridge, England) in a humidified box. The following day, the samples were incubated with the appropriate biotinylated secondary antibodies for 30 min at room temperature. After washing, the sections were visualized using diaminobenzidine (Boster Biological Technology, Wuhan, China), and counter-stained with hematoxylin, dehydrated using a series of increasing concentrations of alcohol solutions and xylene, and sealed with cover slides. Images were captured under a light microscope (magnification 40×).

Observing paraffin sections and counting stained cells by visual under optical microscope, then calculate percentage. Based on the percentage of stained cells occupying all cancer cells in the tissue samples, percentage staining scores were defined as follows: (i) 0, < 10%; (ii) 1, 10–25%; (iii) 2, 25–50%; (iv) 3, 50–75%; and (v) 4, > 75%; intensity scores of staining were divided into four grades: (i) 0, no staining; (ii) 1, light brown; (iii) 2, brown; and (iv) 3, dark brown. Staining positivity was evaluated using IHC scores, which were defined as: IHC score = percentage score × intensity score. Thus, based on the IHC scores, FZD7 expression was classified into four grades: (i) IHC score ≤ 3, negative; (ii) IHC score > 3 and ≤ 6, weak; (iii) IHC > 6 and ≤ 9, moderate; and (iii) IHC > 9, strong.

### Cell culture, spheroid-formation culture assay

The human pancreatic adenocarcinoma cell lines AsPC-1, Capan-2, PANC-1, and SW1990 were obtained from the American Type Culture Collection (ATCC, Rockville, Md.). All cell lines were cultured in DMEM (Gibco; Thermo Fisher Scientific, Inc. Waltham, Mass.) with 10% FBS (MRC Biotechnology Co. Ltd.; cat. no. CCS30009.02. Cincinnati, Ohio) and 100 U/ml penicillin at 37 °C in a humidified incubator with 95% air and 5% CO_2_.

When the adherent PANC-1 cells were 90% confluent, they were digested and resuspended, and the concentration was adjusted to 1 × 10^4^ cells/ml. The cells were then inoculated into complete MammoCult™ Human Medium (Stemcell Technologies, Inc. Vancouver, Canada) using 6-well ultra-low attachment surface polystyrene culture plates (Corning. Inc. Corning, N.Y.). After 15 days, we observed and imaged the morphology and quantity of the pancreatic cell spheres using an inverted light microscope.

### Western blot

Pancreatic adenocarcinoma Cells were lysed using cold RIPA lysis buffer (Beyotime, Boston, Mass), and lysates were centrifuged at 10,000×*g* for 30 min at 4 °C, retaining the supernatants. The BCA Protein Assay Kit (Beyotime) was used to quantify the total protein concentration. Total proteins were loaded on an 8% SDS-gel, resolved using sodium dodecyl sulfate polyacrylamide gel electrophoresis (SDS-PAGE), and then transferred to a polyvinylidene fluoride film (PVDF) membrane. Membranes were blocked in 5% non-fat dried milk in TBST for 2 h and then incubated with the specific primary antibodies overnight, using GAPDH as the loading control. Membranes were subsequently incubated with horseradish peroxidase-coupled secondary antibodies for 2 h. SuperSignal Chemiluminescent Substrates (Thermo Fisher Scientific, Inc., Waltham, Mass.) were then added to the membranes and imaged using an imaging system. The gray values of the bands were normalized to the respective GAPDH bands. The primary antibodies used were as follows: FZD7 (1:1000; Abcam, cat. no. ab64636), active β-catenin (1:1000; Cell Signaling Technology, Inc.; cat. no. 8814, Danvers, Mass.), and GAPDH (1:10,000; ProteinTech Group, Inc.; cat. no. HRP-60004. Chicago, Ill.).

### RNA interference and transfection

Small interfering RNAs (siRNAs) targeting FZD7 and control siRNAs were provided by KeyGen Biotech Co. Ltd (Nanjing, China). PANC-1 cells were seeded and cultured in 6-well plates, and when the cells were 60–80% confluent, they were added to the medium using Lipofectamine™ 3000 reagent (Invitrogen. Inc., Carlsbad, Calif.), according to the manufacturer’s protocol. After 48 h, transfected cells were used in the subsequent experiments. The sequences of the siRNAs targeting FZD7 were as follows: siFZD7-1, 5-GTTCGTCTACCTCTTCATA-3; siFZD7-2, 5-AGTACCTGATGACCATGAT-3; and siFZD7-3, 5-AGCCGTACCACGGAGAGAA-3′. Western blot was used to screen for effective siRNA.

PANC-1 cells were transfected with short hairpin (sh)RNA lentiviruses (KeyGen Biotech Co., Ltd) targeting FZD7 (5-GTTCGTCTACCTCTTCATA-3), and the transfected cells were selected using 2 μg/ml puromycin (Invitrogen; Thermo Fisher Scientific, Inc.) for 48 h. The transfected cells showing healthy growth were maintained in DMEM supplemented with 10% FBS and 2 μg/ml puromycin.

### Wound healing experiment

About 5 × 10^5^ PANC-1 cells per well was seeded in the six well plate for wound healing experiment. A pipette tip was used to scratch the cell monolayer when the adherent cells were 70–90% confluent. The remaining cells were cultured for 24–72 h in serum-free medium. The culture medium was changed every 2–3 days, as usual. An inverted microscope was used to capture images.

### Cell invasion experiment

When the adherent PANC-1 cells were 80–90% confluent, the cells were digested and resuspended in serum-free medium, and the concentration was adjusted to 1 × 10^5^ cells/ml and 0.2 ml of the cell suspension (2 × 10^4^ cells) was added to the upper chamber of the transwell plates (Corning., Inc). In the lower chamber, 0.5 ml medium with 10% FBS was added to promote cell movement through the pores of the membrane. After 48 h, the remaining cells in the upper chamber were cleaned using a cotton swab. Migrated cells were fixed in paraformaldehyde solution for 15 min and stained with crystal violet. Images were captured using an inverted microscope.

### Immunofluorescence staining

PANC-1 cells were seeded on cover slips for 24 h. After the cells were 5–10% confluency, they were fixed in 4% paraformaldehyde solution, and then permeabilized in PBS containing 1% Triton-100 for 10 min. Cells were blocked with 2% BSA prepared in PBS for 10 min and then incubated with primary antibodies (active β-catenin; 1:800, Cell Signaling Technology, Inc.; cat. no. 8814) overnight at 4 °C. Subsequently, cells were incubated with Alexa Fluor 488 goat anti-rabbit IgG(H+L)-conjugated secondary antibodies (1:1000) for 1 h. Cell nuclei were stained with DAPI (Beyotime) for 10 min in the dark. Fluorescence was visualized using a confocal laser-scanning microscope (Leica Laser Technik GmbH. Wetzlar, Germany).

### Flow cytometry assay

Adherent PANC-1 cells (80–90% confluent) were collected and centrifuged. The concentration of cells was adjusted to 1 × 10^6^ cells/ml. For analysis of stemness markers, cells (1 × 10^6^) were labeled with PE-conjugated CD24 (BD Pharmingen™, cat. no. 555428; BD Biosciences, East Rutherford, N.J.), and APC-conjugated anti-CD44 (BD Pharmingen™, cat. No. 559942; BD Biosciences.) and incubated at 4 °C in the dark for 30 min. Cells were then centrifuged and resuspended in PBS at a concentration of 1 × 10^8^/ml, and subsequently sorted on a flow cytometer (BD Accuri C6 Plus; BD Biosciences).

### TGF-β1 induced EMT

For cell culturing about 5 × 10^5^ PANC-1 cells per well was seeded in the six well plate. When cells was well grown and > 80% confluency, we change culture medium to a serum-free culture medium with 10 ng/ml TGF-β1. All process were performed in a humidified 5% CO_2_ atmosphere at 37 °C and we observed cells frequently and confirmed cells being alive.

### Statistical analysis

Data are presented as mean ± standard of at least three independent experiments. The correlation between FZD7 IHC staining and basic clinical information of patients was analyzed using the χ^2^ test. Kaplan–Meier analysis was performed, followed by log-rank test for survival and hepatic metastasis time analysis using GraphPad Prism version 7.0 (GraphPad Software, Inc., San Diego, Calif.). Continuous data were compared using a Student’s t-test for comparison between the two groups. Statistical significance was set at *P* < *0.05*.

## Results

### High FZD7 expression correlates with poor survival in pancreatic cancer

**Fig. 1:**
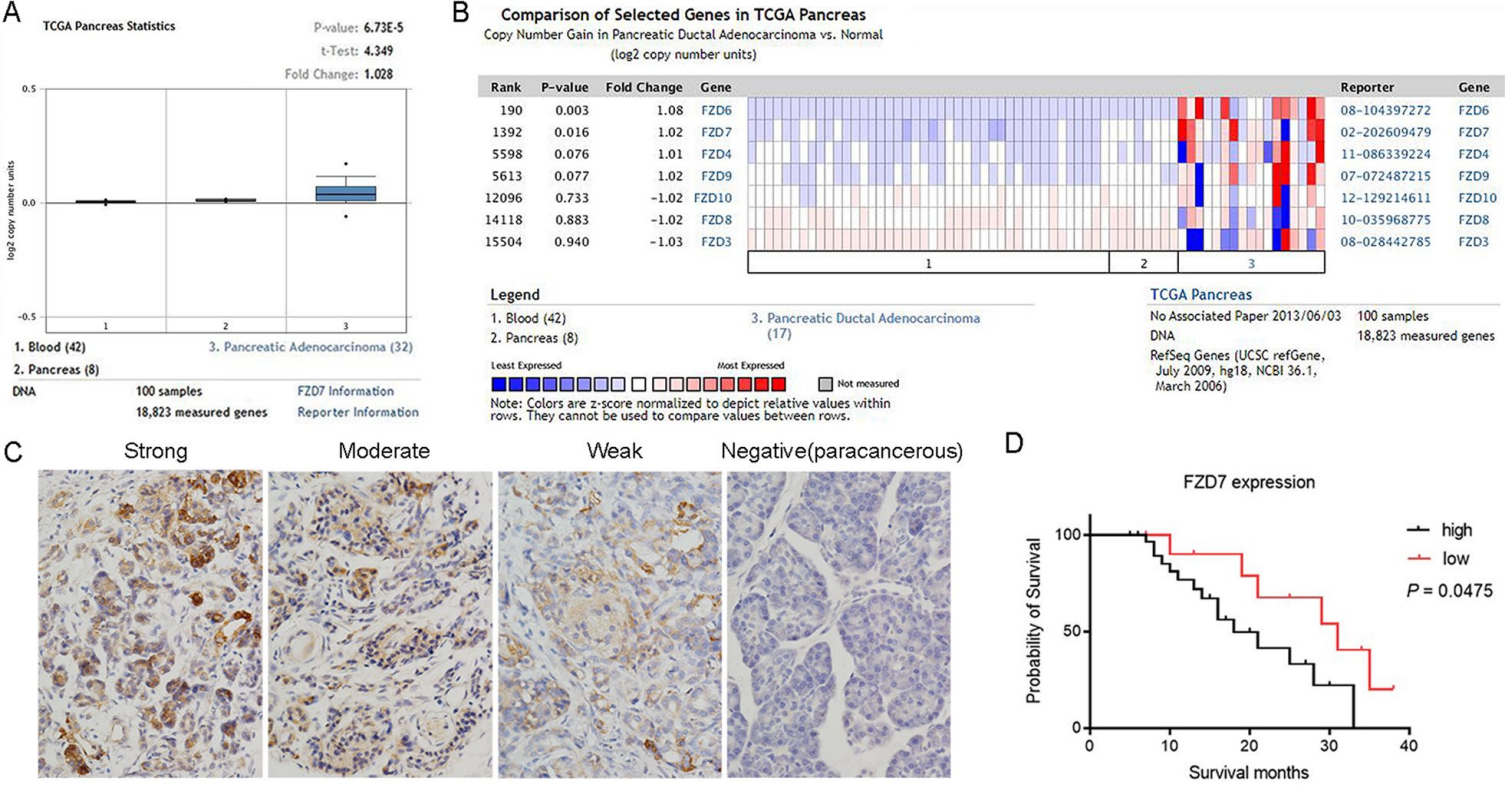
Querying the ONCOMINE database showed that FZD7 expression was significantly higher in pancreatic adenocarcinoma compared with normal pancreatic tissue, a representative result is shown in (**A**); among the different Frizzled Receptors, FZD7 was significantly over-expressed in pancreatic ductal adcarcinoma compared with other Frizzled receptors (**B**). FZD7 expression in cancer tissues was detected in PDAC tissue samples by IHC, which stained darker than paracancerous tissues (**C**). Kaplan–Meier assay showed that high expression of FZD7 was associated with low survival probability, *P* < 0.05 (**D**)

Most datasets from the ONCOMINE database suggested that FZD7 expression was significantly higher in pancreatic cancer than in normal pancreatic tissue (Fig. 1A). In the ONCOMINE database, FZD7 was the most overexpressed Frizzled receptor in pancreatic adcarcinoma (Fig. 1B).

A total of 42 surgical samples of PDAC were included in this study. Frizzled receptor 7 expression was confirmed by immunohistochemical staining of PDAC tissue sections (Fig. 1C), which expression in cancer tissues was significantly higher than that in paracancerous normal pancreatic tissues. Immunohistochemical assay showed that 31/42 cancer tissue samples (73.81%) were positive for FZD7 and 8/42 paracancerous tissues samples (16.67%) were positive for FZD7, with mean IHC scores of 8.2 ± 3.5 and 2.7 ± 3.2, respectively (*P* < 0.05). We analyzed the survival probability of 42 cases based on FZD7 expression, and the results showed that the survival probability of FZD7 in the high expression group was lower than that in the low expression group (*P* < 0.05) (Fig. 1D).

### Patients with high level of FZD7 expression suffered earlier hepatic metastasis

**Fig. 2:**
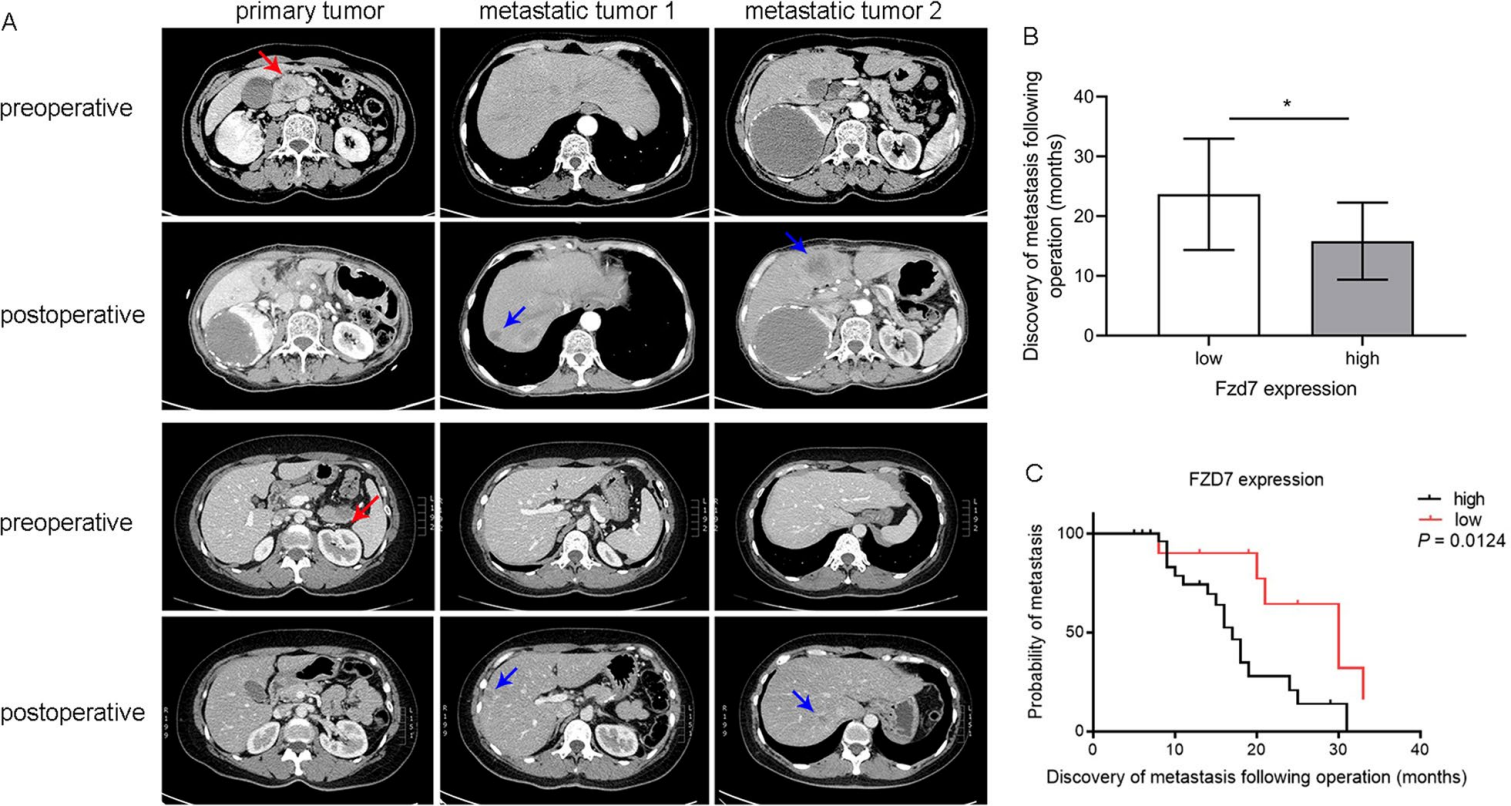
Postoperative hepatic metastasis was confirmed by enhanced CT detection, red arrow tagging primary lesion, blue arrow tagging metastatic tumor (**A**); follow-up assay observed that the mean time of hepatic metastasis was 23.7 ± 9.3 months for 17 of 31 pancreatic cancer patients with high FZD7 expression, while 15.8 ± 6.4 months for 6 of 11 pancreatic cancer patients with low FZD7 expression, *P < 0.05, independent Student’s t-test (**B**); the monthly cumulative metastasis probability of the low FZD7 expression group was lower than that of the high FZD7 expression group, *P* < 0.05, Kaplan–Meier analysis (**C**)

Patients with PDAC were followed up after surgery (radical pancreas body and tail splenectomy or radical pancreaticoduodenectomy), and the liver was examined by ultrasound once a month. Hepatic enhanced CT detection was performed immediately after space-occupying lesions were found in the liver (Fig. 2A), and the time at which hepatic metastasis was initially detected was recorded. During the follow-up, 23 cases of hepatic metastasis were found, including six cases with low FZD7 expression and 17 cases with high FZD7 expression. The time of hepatic metastasis in the low expression group was significantly later than that in the high expression group (Fig. 2B). In addition, the monthly cumulative metastasis probability of the low FZD7 expression group was significantly lower than that of the high FZD7 expression group (Fig. 2C).

### High FZD7 expression promotes EMT of PDAC cells

By Western blot, we confirmed that FZD7 was expressed in AsPC-1, Capan-2, PANC-1 and SW1990 cells to different degrees, of which PANC-1 was derived from pancreatic ductal adenocarcinoma. Pancreatic ductal adenocarcinoma accounts for 90% pancreatic cancer malignancies (Jemal et al. 2008), and our study was based on pancreatic ductal adenocarcinoma, so we selected PANC-1 cells for the other vitro cell experiments. Moreover, our results showed that FZD7 was significantly expressed in PANC-1, which was conducive to further down-regulation of FZD7 expression to observe cell phenotype and functional changes.

**Fig. 3:**
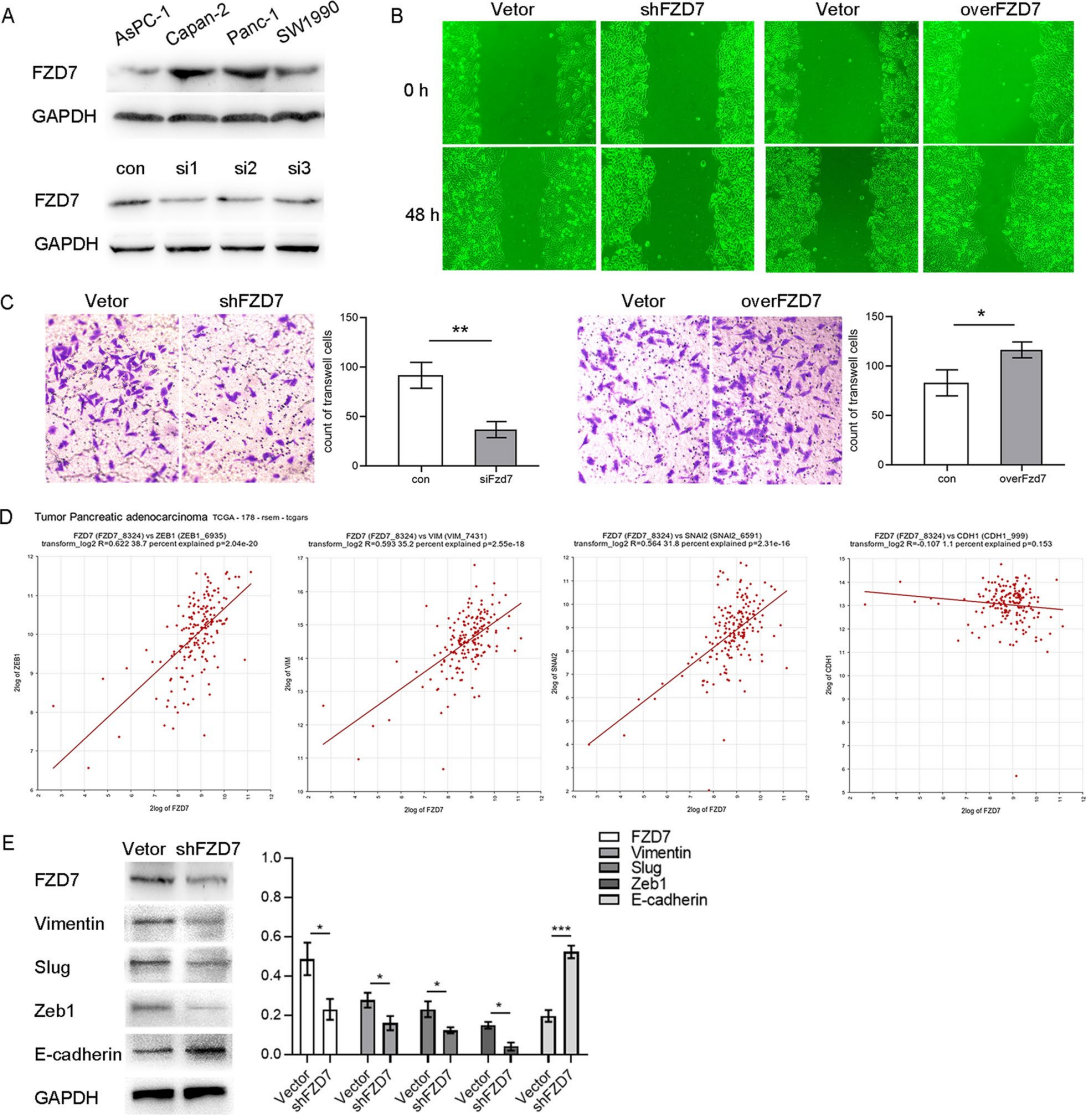
PANC-1 was selected for experimentation in vitro, and the effective siRNA was screened by Western blot (**A**). Silencing FZD7 suppressed PANC-1 cell migration and invasion in Wound healing and Transwell assays (**B**); whereas FZD7 over-expression promoted PANC-1 cell migration and invasion in Wound healing and Transwell assay (**C**). In human PDAC, FZD7 gene expression was significantly positively correlated with that of the mesenchymal-related genes, *ZEB1*, *VIM*, *SNAIL2*, with Pearson co-efficients of 0.622 (*P* = 2.04e^−20^), 0.593 (*P* = 2.55e^−18^), and 0.564 (*P* = 2.31e^−16^), respectively, and non-significantly negatively correlated with the epithelial-related gene *CDH1* (*P* = 0.153) (**D**). In PDAC cells, after the expression of FZD7 was down-regulated by siRNAs, the expression of ZEB1, Vimentin and Slug proteins were all reduced with statistical significance (**E**). Significant, *P < 0.05, **P < 0.01, ***P < 0.001, Student’s t-test

We downregulated FZD7 expression in PANC-1 cells using siRNA, we selected nucleic acid sequences of efficient siRNA chain by Western blot to construct lentiviral vectors carrying shFZD7-RNA (Fig. 3A), and found that silencing FZD7 inhibited PANC-1 cell migration and invasion ability in wound healing and transwell assays (Fig. 3B), whereas FZD7 overexpression promoted PANC-1 cell migration and invasion in wound healing and Transwell experiments (Fig. 3C). To investigate the association of FZD7 with the mesenchymal phenotype in PDAC, we first queried the R2 gene database. We found that in human PDAC, the level of FZD7 gene expression was positively correlated with mesenchymal-related genes, such as *VIM*, *SNAIL2*, *ZEB1*, and negatively correlated with epithelial-related genes, *CDH1* (Fig. 3D). Moreover, silencing FZD7 expression of PANC-1 in vitro, resulted in decrease of the levels of Vimentin, Zeb1, and Slug (Fig. 3E).

### FZD7 strengthens stemness of PDAC cells

**Fig. 4:**
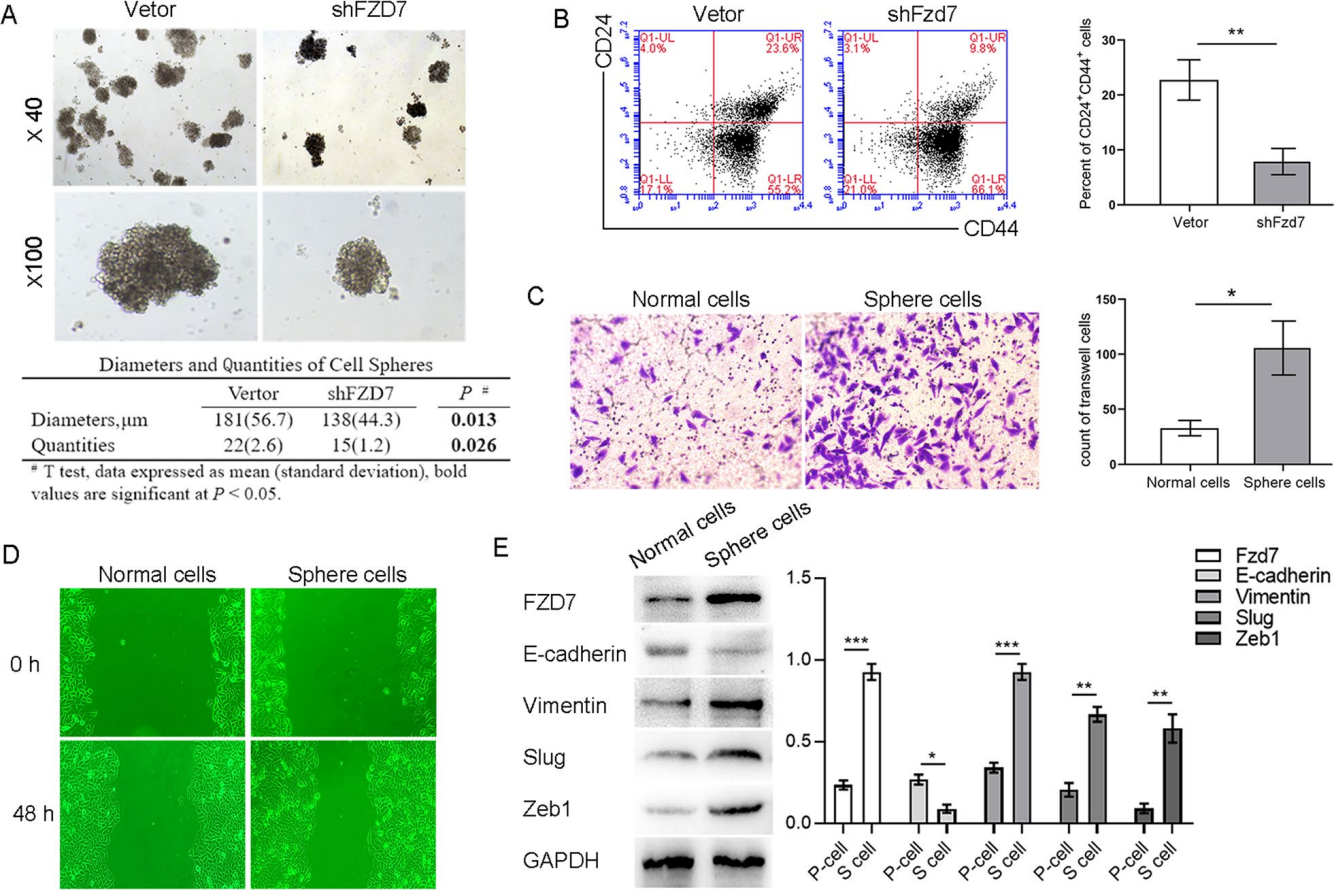
After silencing FZD7 expression using lentiviral shRNAs, the counts and the diameters of cell spheres were significantly decreased during suspension culture (**A**), and the proportion of CD24^+^CD44^+^ cells in PANC-1 cell line was reduced from 23.6 to 9.8% (**B**). The spheroid-forming cell migration distance was larger than that in normal adherent culture cells (**C**), and the proportion of Transwell cells was higher than that of normal adherent culture cells (**D**). The spheroid-forming cell expressed higher levels of mesenchymal phenotype (**E**). Significant, *P < 0.05, **P < 0.01, ***P < 0.001, Student’s t-test

Cell spheroid-forming assays are commonly used to assess the self-renewal and differentiation potential of putative stem cells growing in non-adherent conditions, and have been successfully applied to CSC biology studies (Dontu et al. 2003). We found that after silencing FZD7 expression using lentiviral shRNAs in PANC-1 cells, the number and diameter of cell spheres were significantly decreased during suspension culture (Fig. 4A). Flow cytometry revealed that the percentage of CD24^+^CD44^+^ subsets in PANC-1 cells was also reduced (Fig. 4B). In the wound healing and transwell experiments, spheroid-forming cells exhibited stronger invasion and migration ability (Fig. 4C, D) and a higher level of EMT relating phenotype than normal adherent cells (Fig. 4E).

### FZD7 acts via the canonical WNT/β-catenin pathway

**Fig. 5:**
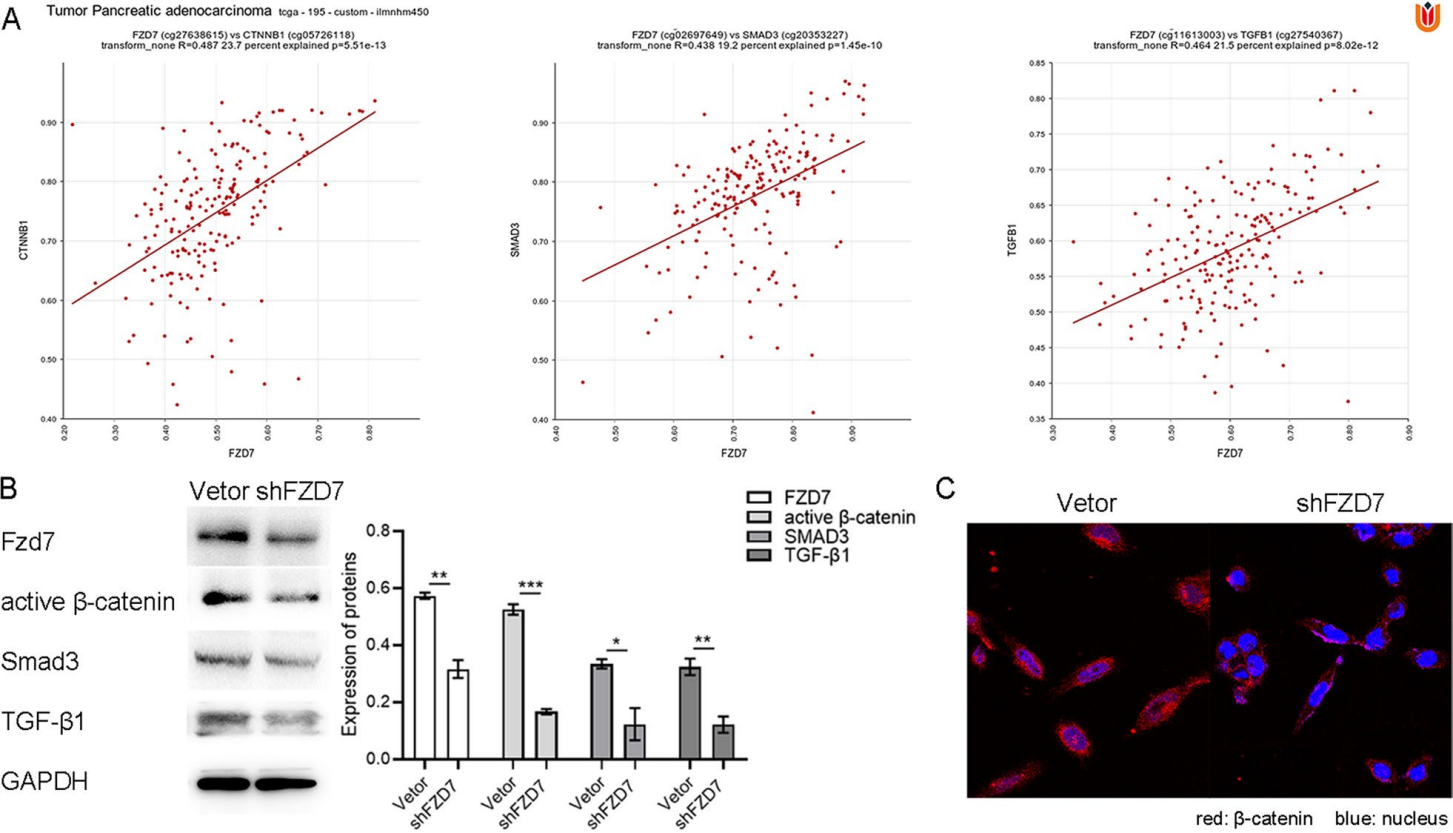
FZD7 was positively correlated with the expressions of CTNNB1, SMAD3 and TGF-β1 at the gene level, with Pearson co-efficient values of 0.487 (*P* = 5.51e^−13^), 0.438 (*P* = 1.45e^−10^), and 0.464 (*P* = 8.02e^−12^) respectively (**A**). Western blotting assay confirmed that the level of active β-catenin, SMAD3 and TGF-β1 was decreased after FZD7 silenced (**B**). The reduction of active β-catenin after FZD7 silenced inside and outside of the nucleus was detected using immunofluorescence (**C**). Significant, *P < 0.05, **P < 0.01, ***P < 0.001, Student’s t-test

Results from the R2 gene database indicated that *FZD7* expression positively correlated with expression of *CTNNB1*, *TGF-β1*, and *SMAD3* at the gene level (Fig. 5A). Western blot confirmed that the levels of active β-catenin, TGF-β, and SMAD3 were decreased after the protein expression of FZD7 was decreased (Fig. 5B). Using immunofluorescence, we observed that the levels of active β-catenin inside and outside of the nucleus were significantly reduced after the expression of FZD7 was morphologically downregulated in PANC-1 cells (Fig. 5C).

### FZD7 can mediate TGF-β1 inducing EMT and CSC phenotype protein expression

**Fig. 6:**
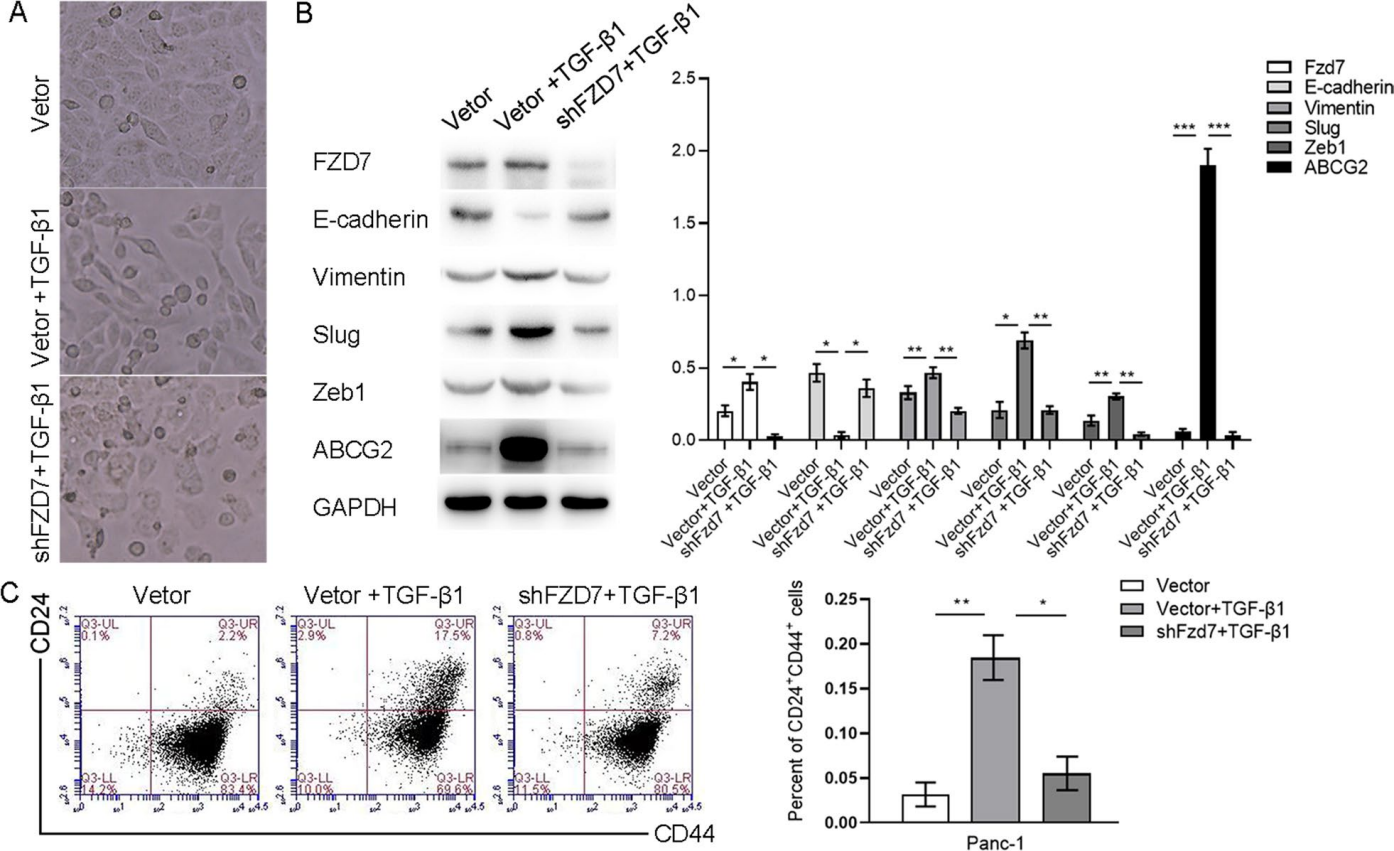
After treatment of 10 ng/ml TGF-β1 for 48 h, PANC-1 cells acquired a fusiform and elongated morphology, while shFZD7-PANC-1 acquired no morphological change (**A**). Western blot showed that after the treatment of TGF-β1, Vimentin, Slug, Zeb1, ABCG2 expression were increased, similarly, TGF-β1 could not change these phenotype protein expression levels of shFZD7-PANC-1 (**B**). CD24^+^CD44^+^ cells population representing CSC subsets were increased after treatment of TGF-β1, when FZD7 expression was silenced, this effect was attenuated (**C**). Significant, *P < 0.05, **P < 0.01, ***P < 0.001, Student’s t-test

Cells undergone EMT tend to be fusiform in morphology, and obtain enhanced migration and invasion ability in biological behavior (Yoshida et al. 2020). After treatment of 10 ng/ml TGF-β1 for 48 h, PANC-1 cells acquired a fusiform and elongated morphology, while shFZD7-PANC-1 acquired no morphological change (Fig. 6A). Western blot testing showed that after the treatment of TGF-β1, Vimentin, Slug, Zeb1 expression was increased, similarly, TGF-β1 could not change these phenotype expression levels of shFZD7-PANC-1 (Fig. 6B). CSC phenotype protein ABCG2 and CD24^+^CD44^+^ cells population representing CSC subsets were increased after treatment of TGF-β1, however when FZD7 expression was silenced, this effect was attenuated (Fig. 6B, C).

## Discussion

Cells experienced the EMT procedure exhibit loosened tight junctions and cell-to-cell adhesion to be ready for migration (Iqbal et al. 2016; Tsubakihara and Moustakas 2018). Elevated numbers of CTCs enhanced tumor aggressiveness (Hermann et al. 2014). The quantity of CTCs in the preoperative blood circulation directly affects the occurrence of postoperative hepatic metastasis and survival time, many studies have used CTC as an independent factor to evaluate the stage and prognosis of malignant tumors: whether there are three or more CTCs in 4 ml whole blood discriminated between localized and metastatic stage (Ankeny et al. 2016).

Among the 42 PDAC patients TNM staged I–II without vascular invasion and liver metastasis, postoperative immunohistochemical assay confirmed 31 cases of FZD7 positive. Follow-up studies revealed that hepatic metastasis occurred earlier in the group with a high level of FZD7 expression. We also found that the expression of FZD7 was positively associated with migration and invasion ability in vitro. Therefore, we think that high FZD7 expression can promote EMT of pancreatic cancer cells, more cancer cells had undergone EMT and disseminated into the blood circulation before obvious hepatic metastasis tumor occurred, so that the chance of hepatic metastasis was significantly higher than that in patients with low FZD7 expression.

WNT signaling pathway has been reported to promote EMT by upregulating the transcription factors Slug and Twist (DiMeo et al. 2009). R2 database retrieval analysis results show that FZD7 expression had a high Pearson correlation coefficient with the level of the mesenchymal phenotypic regulator, *Vimentin*, as well as the EMT-related transcription factors *Snail2* and *Zeb1*. In addition, western blot also confirmed that the levels of Vimentin, Slug, and Zeb1 proteins decreased after the downregulation of FZD7 protein expression. Therefore, FZD7 may play an important role in the EMT process of pancreatic cancer.

Spheroid-forming cultures of human PDAC cells enriched CSCs, and showed increased Snai1 expression levels in sphere cells which representing CSC subsets (Wang et al. 2016). While in the spheroid-forming culture process of this study, silencing FZD7 expression caused the number and size of cell spheres, and the proportion of CD24^+^CD44^+^ cell subset to reduce, suggesting that ability of CSC proliferation was attenuated. The migration and invasion ability of spheroid-forming cells in the Wound healing assay and Transwell experiments were stronger than those of normal adherent cells and expressed higher levels of EMT-related protein molecules, such as Vimentin, Slug, Zeb1, which means that the morphology and biological characteristics of CSC are closer to mesenchymal cells. CTCs have been demonstrated to have both tumor-initiating cells and mesenchymal cells traits (Poruk et al. 2017), and the association between EMT and CSCs has been demonstrated in multiple types of human malignant tumors (Shibue and Weinberg 2017). In murine PDAC models, CTCs formed after EMT showed obvious CSC characteristics and could be colonized in the liver to form metastatic carcinoma easily (Rhim et al. 2012). A study on the breast cancer cells first indicated that undergoing EMT can promote mesenchymal and stemness properties of breast cancer cells (Mani et al. 2008). All these results reveal the subtle relationship between EMT and CSC. It is possible that EMT is a process of reverse differentiation into stem cells.

It is currently believed that CSCs are more resistant to chemotherapy than normal tumor cells (Rao and Mohammed 2015; Valle et al. 2018); Our previous study also showed that FZD7 can synergize with Wnt7b to increase the proportion of PDAC CSCs and enhance drug resistance of pancreatic cancer cells (Zhang et al. 2021). Therefore, patients with high levels of FZD7 may also be more resistant to postoperative chemotherapy and other drug treatments, indirectly affecting the occurrence of liver metastasis.

A reduction of β-catenin was observed in FZD7-silenced cells, followed by downregulation of c-Myc and cyclin D1, which are target genes of the canonical WNT pathway (Li et al. 2018). Accumulating evidence has confirmed that FZD7 can mediate the activation of the canonical WNT/β-catenin signaling in breast, colorectal, and hepatocellular carcinoma (King et al. 2012). The canonical WNT signaling pathway mediated by FZD7 can promote breast cancer stem cell activity (Chakrabarti et al. 2014). Our experiment also confirmed that the levels of active β-catenin, TGF-β1, and SMAD3 decreased after the downregulation of FZD7 expression. Therefore, we assume that FZD7 plays a role through the canonical WNT pathway, and that the TGF-β/SMAD3 pathway could be associated with the regulation of the FZD7/β-catenin pathway in the malignant biological behavior of pancreatic cancer cells.

TGF-β1 induced significant mesenchymal morphological changes in PANC-1 cells in vitro, and the levels of mesenchymal proteins and CSC phenotypic proteins increased at the same time. However, silencing FZD7 expression can attenuate this effect. Therefore we speculated that TGF-β/SMAD3 signaling pathway can not only induce EMT, but also regulate CSC population. Some scholars have proposed that the origin and development of CSC is regulated by the EMT process (Islam et al. 2015a, b). FZD7 as the receptor of WNT signaling pathway can also mediate the activation of TGF-β/SMAD3 signaling pathway. For example, there has been many similar results reported, in breast cancer, hepatic carcinoma, gastric carcinoma, skin cancer and malignant glioma, TGF-β1 can improve renewal ability of CSC and sustain the basic function of CSC by different mechanisms (Claudia et al. 2016); especially in breast cancer, TGF-β1 can act synergistically with WNT signaling pathway to induce EMT and sustain mesenchymal phenotype and CSC state. Blocking TGF-β1 function and downstream signaling pathway can lead to migration ability and CSC self-renewal ability reduced in vitro, tumorigenic potential reduced and incidence of developing metastases in mouse models in vivo (Scheel et al. 2011).

## Conclusion

In pancreatic ductal adenocarcinoma patients, high FZD7 expression associated with earlier hepatic metastases. High FZD7 expression can promote EMT by amplifying CSC subsets through the canonical WNT signaling pathway, CSC possess high degree of EMT. FZD7 can participate in TGF-β/SMAD3 signaling pathway to promote EMT also.

## Data Availability

The data that support the findings of this study are available from the corresponding author upon reasonable request.
